# FEW OLDER DRIVERS TAKE REFRESHER COURSES: IDENTIFICATION OF FACILITATORS AND BARRIERS

**DOI:** 10.1093/geroni/igae098.1312

**Published:** 2024-12-31

**Authors:** Michel Bedard, Hillary Maxwell, isabelle Gelinas, Gary Naglie, Brenda Vrlkjan, Michelle Porter, Holly Tuokko, Shawn marshall

**Affiliations:** Lakehead University, Thunder Bay, Ontario, Canada; Lakehead University, Thunder Bay, Ontario, Canada; McGill University, Montreal, Quebec, Canada; Baycrest Hospital, Toronto, Ontario, Canada; McMaster University, Hamilton, Ontario, Canada; University of Manitoba, Winnipeg, Manitoba, Canada; University of Victoria, Victoria, British Columbia, Canada; Ottawa Hospital, Ottawa, Ontario, Canada

## Abstract

Older drivers may benefit from driver refresher courses, particularly from courses with on-road training. Yet, the uptake of refresher courses and the factors associated with the decision to enroll is poorly documented. We examine these issues using the Candrive prospective cohort (recruitment stated in 2009; N = 927), a representative sample of older Canadian drivers. We operationalized our outcome variable as having taken any non-mandatory refresher course within the last 10 years (yes/no). We developed a conceptual model that includes sociodemographic factors (e.g., age), health-related factors (e.g., medical conditions), and driving-related factors (e.g., driving comfort), and identified the contribution of these factors using logistic regression. Participants’ mean age was 76.21 (SD=4.85) and 576 (62.1%) were males. Ninety-eight participants (10.6%) reported having taken a non-mandatory refresher course within the last 10 years; roughly half reported the course included an on-road component. The multivariable regression model (-2 log likelihood = 518.41, p <.001, Nagelkerke R-square =.17) identified six of 13 variables entered into the model as statistically significant. The odds of having taken a course differed by province of residence, and was higher with increasing age, if their driving was important for others, were a volunteer, and spoke to a physician about driving, but was lower if they enjoyed driving. Our results indicate that the uptake of refresher courses is limited but is associated with potentially modifiable factors. Deepening our understanding of these facilitators and barriers will help support older adults to drive safely for longer.

